# Venous thromboembolism prophylaxis practice and its association with outcomes in Australia and New Zealand burns patients

**DOI:** 10.1093/burnst/tkaa044

**Published:** 2021-02-11

**Authors:** Lincoln M Tracy, Peter A Cameron, Yvonne Singer, Arul Earnest, Fiona Wood, Heather Cleland, Belinda J Gabbe

**Affiliations:** 1 School of Public Health and Preventive Medicine, Monash University, 553 St Kilda Road, Melbourne, Victoria, 3004, Australia; 2 Emergency and Trauma Centre, The Alfred Hospital, Commercial Road, Melbourne, Victoria, 3004, Australia; 3 Victorian Adult Burns Service, Alfred Hospital, 55 Commercial Road, Melbourne, Victoria, 3004, Australia, Australia; 4 Registry Sciences Unit, Department of Epidemiology and Preventive Medicine, Monash University, 553 St Kilda Road, Melbourne, Victoria, 3004, Australia; 5 Biostatistics Unit, Department of Epidemiology and Preventive Medicine, Monash University, 553 St Kilda Road, Melbourne, Victoria, 3004, Australia; 6 Burn Injury Research Unit, University of Western Australia, 35 Stirling Highway, Perth, Western Australia, 6009, Australia; 7 Health Data Research UK, Swansea University Medical School, Swansea University, Singleton Park, Swansea, UK

**Keywords:** Burn injury, Venous thromboembolism, Prophylaxis, Australia, New Zealand

## Abstract

**Background:**

Patients with burn injuries are considered to have an increased risk of venous thromboembolism (VTE). While untreated VTEs can be fatal, no studies have examined chemoprophylaxis effectiveness. This study aimed to quantify the variation in prevalence of VTE prophylaxis use in patients in Australian and New Zealand burns units and whether prophylaxis use is associated with in-hospital outcomes following burn injury.

**Methods:**

Admission data for adult burns patients (aged ≥16 years) admitted between 1 July 2016 and 31 December 2018 were extracted from the Burns Registry of Australia and New Zealand. Mixed effects logistic regression modelling investigated whether VTE prophylaxis use was associated with the primary outcome of in-hospital mortality.

**Results:**

There were 5066 admissions over the study period. Of these patients, 81% (n = 3799) with a valid response to the VTE prophylaxis data field received some form of VTE prophylaxis. Use of VTE prophylaxis ranged from 48.6% to 94.8% of patients between units. In-hospital death was recorded in <1% of patients (n = 33). After adjusting for confounders, receiving VTE prophylaxis was associated with a decrease in the adjusted odds of in-hospital mortality (adjusted odds ratio = 0.21; 95% CI, 0.07–0.63; *p* = 0.006).

**Conclusions:**

Variation in the use of VTE prophylaxis was observed between the units, and prophylaxis use was associated with a decrease in the odds of mortality. These findings provide an opportunity to engage with units to further explore differences in prophylaxis use and develop future best practice guidelines.

Highlights

- Burn patients are thought to have an increased risk of venous thromboembolism (VTE).
- While VTEs are associated with an increased risk of death, less is known about the use of VTE prophylaxis use in burn patients.
- Four out of five patients received VTE prophylaxis during their admission, but there was considerable variation in prophylaxis administration between burn units.
- After adjusting for confounding factors, VTE prophylaxis use was associated with a decrease in the odds of in-hospital mortality.
- We will engage with burn units to further explore differences in VTE prophylaxis use and develop best practice guidelines.

## Background

People with burn injuries are theoretically at an increased risk for venous thromboembolism (VTE) due to systemic hypercoagulability, intimal damage and prolonged periods of bed rest [1–3]. Other factors known to elevate the risk of VTE include increased age, body mass index and total body surface area (TBSA) of the burn [4]. There is a substantial economic burden associated with VTE. MacDougall *et al.* reported that the annual median total healthcare cost for US patients with an isolated deep vein thrombosis or pulmonary embolism to be $15,843 [5]; a number that would increase with the associated costs of burn injury. More locally, an Australian report cited the financial cost of VTE in 2008 as $1.7 billion [6].

Despite the aforementioned risk factors, VTEs are rare in burn patients [2]. Using data from the American Burn Association’s National Burn Repository (NBR) for more than 30,000 patients between 1995 and 2007, Pannucci *et al.* reported the incidence of VTE in thermally-injured burn patients to be 0.6% [7]. However, in other studies the incidence of VTE in burn patients has been reported to be as high as 60% [8]. Such substantial variation in the reported incidence can be attributed to differences in the nature of the study (e.g. retrospective versus prospective), the specific inclusion criteria (e.g. the severity of the burn) and the screening modality used [9]. Discrepancies in the reported incidence of VTE aside, the importance of VTE prevention cannot be understated; VTE is associated with a three-fold increase in risk of death after controlling for baseline risk factors and other relevant comorbidities [7].

Mechanical and pharmacological anticoagulation prophylactic methods can minimize the risk and incidence of VTE events (and subsequent death). Common non-pharmacological VTE prophylaxis measures include early mobilization of the patient [10], intermittent pneumatic compression [11] and graduated compression stockings [12]. In addition, there are a range of pharmacological anticoagulation therapies available [13]. However, there has been debate amongst the burns community as to the effectiveness of VTE chemoprophylaxis in burn patients [1, 14]. In addition, there has been limited research on the effectiveness of VTE prophylaxis use [1, 14]. Consequently, as of 2017, there were no universally accepted guidelines for the use of VTE prophylaxis in burn patients [15].

Given its low incidence in burn patients, matters relating to VTE, particularly the effectiveness of potential prophylaxis treatments, cannot be studied in depth using case series or small, single-centre studies. Larger-scale, multi-centre studies or studies using repositories, databases or registries are better suited to exploring such infrequently occurring outcome events. These larger data sources allow for better control over confounding variables, amongst other benefits [2]. In 2017, Pannucci *et al.* claimed there had been no randomized controlled trials examining the effectiveness of chemoprophylaxis in thermally injured burn patients [2]. Larger-scale registry studies (both prospective and retrospective) may assist in filling the gap in research evidence and best practice. This study aimed to quantify the variation in prevalence of VTE prophylaxis use in patients in Australian and New Zealand burn units and determine whether variation is associated with on in-hospital outcomes following burn injury by using a large, bi-national burn injury registry.

## Methods

### Setting and data source

Within Australia and New Zealand, specialist burn care is provided by 17 burn units. This study used data from the Burns Registry of Australia and New Zealand (BRANZ), a collaboration between the Australian and New Zealand Burn Association and the Department of Epidemiology and Preventive Medicine at Monash University. As of July 2016, all 17 specialist Australian and New Zealand burns units were contributing data to the BRANZ. Further information about the registry, such as inclusion and exclusion criteria, have been published elsewhere [16–22].

In July 2016, the BRANZ began collecting data on VTE prophylaxis administration practices with the addition of the following question to the registry: “If the patient is 16 years or older, did they receive anticoagulation prophylaxis?” In terms of analysable data, valid responses to the VTE prophylaxis question included “yes” (indicating that the patient received prophylaxis) and “no” (indicating that the patient did not received prophylaxis). Invalid responses included “not stated/inadequately described” (indicating it was unclear whether or not the patient received prophylaxis), “not applicable” (the designated response for patients under the age of 16 or patients who received end-of-life care on admission) and missing data (i.e. where no response had been entered for the data field). Prescribed medications acknowledged by the BRANZ for VTE prophylaxis include heparin, warfarin and enoxaparin sodium (or trade named versions of these medications). This list assists data collectors to identify if the patient received pharmacological prophylaxis during their admission.

### Participants

Acute admissions data (i.e. the first admission to a BRANZ-contributing hospital with a new burn injury) from adult burn units between 1 July 2016 and 31 December 2018 were extracted from the registry. Only patients aged ≥16 years at the time of their burn injury were included. Intersex patients or patients of indeterminate gender were excluded due to low counts. Patients aged ≥16 years who were admitted to a paediatric burn unit were excluded, as were patients for whom age could not be calculated. Patients who were palliated on arrival, discharged to another BRANZ hospital or who had a hospital length of stay <24 hours were excluded from the study.

### Data management

As injuries to the chest and lower limb have been associated with the development of VTE [23, 24], data fields relating to the body region of injury were extracted from the BRANZ. A burn to the chest or trunk was defined by a “yes” response to the BRANZ chest region data field or a “front”, “back”, or “front and back” response to the BRANZ trunk region field. A burn to the legs or feet was defined by either a “unilateral” or “bilateral” response to one of the following BRANZ data fields indicating the body region of the burn: foot, foot—dorsum, foot—sole and lower limb (excluding foot). A non-fatal VTE event was identified in patients who survived to discharge and had a relevant International Statistical Classification of Diseases and Related Health Problems, 10th Revision, Australian Modification (ICD-10-AM) diagnosis code [25] accompanying their admission data (Table S1). The relevant ICD-10-AM codes were selected through consultation with registry collaborators. The ICD-10-AM codes were also mapped to the Charlson Comorbidity Index (CCI) to define the comorbid status of patients, with a CCI weighting of zero representing no comorbidities [26].

### Statistical analyses

The demographic, event and injury characteristics of patients who did and did not receive VTE prophylaxis were described as frequencies and percentages (for categorical variables) or as the median and interquartile range (for continuous variables). Mann–Whitney *U* and Chi-squared tests were used, as appropriate, to compare the groups. Unadjusted and risk-adjusted funnel plots were generated to compare the variation in VTE prophylaxis administration between units contributing to the registry, as per Spiegelhalter’s method [27]. The risk-adjusted funnel plot included the following covariates: age; gender; primary cause of the burn; natural logarithm transformation of the %TBSA burned; whether the patient had a full thickness burn; whether the patient had a documented inhalation injury; whether the patient sustained a burn to their leg and/or foot; whether the patient sustained a burn to their chest and/or trunk; and whether the patient was admitted to the intensive care unit (ICU). The VTE administration rates and the number of admissions for each unit were calculated using the `funnelcompar' command in Stata, and control limits corresponding to 95% (2 SDs from the mean) and 99.8% (3 SDs from the mean) around the overall mean administration rate were constructed. These limits correspond to a type I error rate of 5% and 0.2%, respectively. Units >3 SDs from the overall mean administration rate were deemed to be outliers. Units were placed into one of the following three groups based on the number of patients treated and their risk-adjusted VTE prophylaxis administration rate: <60% administration, 60–79% administration and >80% administration. In-hospital deaths and non-fatal VTE event data for these three groups were described using frequencies and percentages before being compared using Fisher’s exact test to determine the association between administration rate and study outcomes. A mixed effects binary logistic regression model (accounting for the random effects of the contributing unit) was constructed to further investigate the variation in practice of administering VTE prophylaxis and quantify the amount of variance explained by other site-specific, rather than patient, factors.

Mixed effects logistic regression models (accounting for the random effects of the contributing unit) were constructed to investigate whether there was an association between whether or not the patient received VTE prophylaxis and relevant in-hospital outcomes. The primary outcome of interest was in-hospital mortality, while non-fatal VTE events were the secondary outcome of interest. Unadjusted models were run initially, followed by risk-adjusted models that accounted for true confounders—characteristics that differed between both the patient group and the outcome. Unadjusted and adjusted odds ratios (ORs) and the corresponding 95% CIs are reported for each logistic regression model. All analyses were conducted using Stata Version 14 (StataCorp, College Station, TX, USA). A *p* value <0.05 was considered statistically significant. With the exception of reporting the proportion of patients with a valid and invalid response to the VTE prophylaxis field, patients with an invalid response to the VTE prophylaxis field were excluded from all main analyses.

## Results

### Prevalence of VTE prophylaxis administration

**Figure 1.**
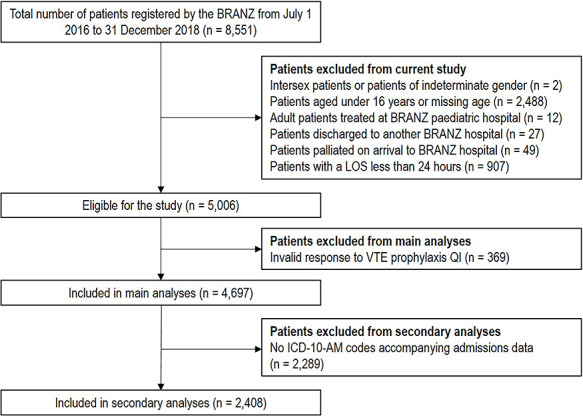
Flow chart for patient inclusion and exclusion in study. *BRANZ* Burns Registry of Australia and New Zealand, *LOS* length of stay, *VTE* venous thromboembolism, *QI* quality indicator, *ICD-10-AM* International Statistical Classification of Diseases and Related Health Problems, 10th Revision, Australian Modification

Figure 1 displays a flowchart of patient inclusion and exclusion for the study. During the period of 1 July 2016 to 31 December 2018, there were 5066 admissions to specialist adult burn units in Australia and New Zealand. Almost all admissions (n = 4697; 92.7%) had a valid response to the VTE prophylaxis data item in the registry. Table S2 contains a comparison of the demographic, event and injury characteristics for patients with and without a valid response to the VTE prophylaxis data item. Of the patients with a valid response, 80.9% of patients (n = 3799) received VTE prophylaxis during their admission.

### Characteristics of patients receiving VTE prophylaxis

Table 1 displays the demographic, event and injury characteristics for patients included in the study. Patients who received VTE prophylaxis were older and had sustained larger and deeper burns compared to patients who did not receive prophylaxis. A greater proportion of patients who received prophylaxis sustained a burn to their chest and/or trunk, leg and/or foot, had documentation of an inhalation injury and were admitted to the ICU. A greater proportion of patients who received prophylaxis had ICD-10-AM codes accompanying their admission data, but there was no association between receiving VTE prophylaxis and CCI weighting groups.

**Table 1:** Demographic, event and injury characteristics for study patients

**Variables**	**No prophylaxis (n = 898)**	**Received prophylaxis (n = 3799)**	***P*-value**
Age, median (IQR) years	38.0 (25.0–53.0)	43.0 (28.0–58.0)	<0.001^a^
Gender			<0.001
Male	611 (68.0%)	2799 (73.7%)	
Female	287 (32.0%)	1000 (26.3%)	
Primary cause of burn injury^b^			<0.001
Flame	345 (38.7%)	1814 (48.0%)	
Scald	271 (30.4%)	992 (26.2%)	
Contact	140 (15.7%)	504 (13.3%)	
Other Cause	136 (15.2%)	473 (12.5%)	
TBSA, median (IQR)^c^	2.0 (1.0–4.1)	4.0 (1.5–9.5)	<0.001^a^
FT TBSA, median (IQR)^d,e^	1.0 (0.5–1.5)	2.0 (1.0–5.0)	<0.001^a^
%TBSA group^c^			<0.001
0–9%	794 (92.8%)	2817 (75.1%)	
10–19%	45 (5.3%)	601 (16.0%)	
≥20%	17 (2.0%)	333 (8.9%)	
Superficial burn^f^			<0.001
No	374 (48.3%)	1930 (56.0%)	
Yes	400 (51.7%)	1517 (44.0%)	
FT burn^g^			<0.001
No	554 (72.9%)	2099 (60.6%)	
Yes	206 (27.1%)	1365 (39.4%)	
Burn to chest or trunk			<0.001
No	707 (78.7%)	2664 (70.1%)	
Yes	191 (21.3%)	1135 (29.9%)	
Burn to legs or feet			<0.001
No	532 (59.2%)	1360 (35.8%)	
Yes	366 (40.8%)	2439 (64.2%)	
Inhalation injury^h^			<0.001
No	867 (97.3%)	3531 (93.4%)	
Yes	24 (2.7%)	250 (6.6%)	
ICU admission^i^			<0.001
No	862 (96.1%)	3264 (85.9%)	
Yes	35 (3.9%)	534 (14.1%)	
ICU LOS, median (IQR) hours^j,k^	30.0 (17.0–62.0)	72.5 (34.4–264.4)	<0.001^a^
ICD-10-AM codes submitted			<0.001
No	544 (60.6%)	1745 (45.9%)	
Yes	354 (39.4%)	2054 (54.1%)	
CCI weight^l^			0.08
0	296 (83.6%)	1623 (79.0%)	
1	43 (12.1%)	286 (13.9%)	
>1	15 (4.2%)	145 (7.1%)	

Data are presented as frequency (percentage) unless otherwise specified. All *p* values are from a Chi-squared test, unless otherwise specified.

*IQR* interquartile range, *TBSA* total body surface area, *FT* full thickness, *ICU* intensive care unit, *LOS* length of stay, *ICD-10-AM* International Statistical Classification of Diseases and Related Health Problems, 10th Revision, Australian Modification, *CCI* Charlson Comorbidity Index.

^a^ *p* values from Mann–Whitney *U* test

^b^Data missing for 22 patients

^c^Data missing for 90 patients

^d^For patients with a full thickness burn

^e^Data missing for 149 patients

^f^Data missing for 476 patients

^g^Data missing for 473 patients

^h^Data missing for 25 patients

^i^Data missing for 2 patients

^j^For patients admitted to the ICU

^k^Data missing for 2 patients

^l^For patients with ICD-10-AM codes

### Variation in VTE prophylaxis administration practices

**Figure 2.**
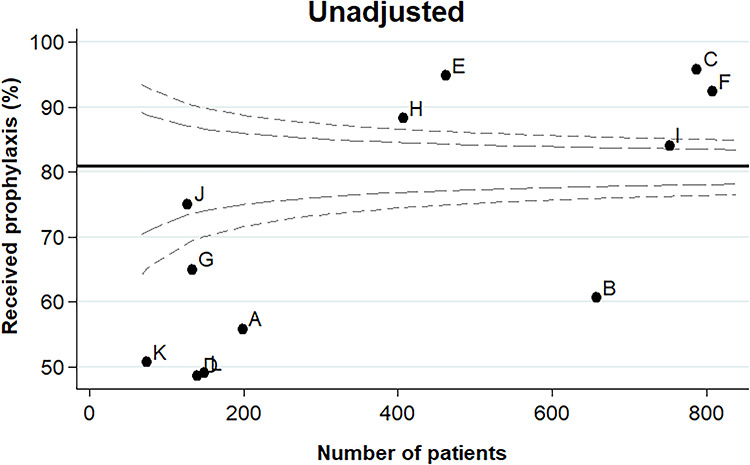
Unadjusted funnel plot for venous thromboembolism (VTE) prophylaxis administration rates across Burns Registry of Australia and New Zealand units. The black line represents the overall mean VTE prophylaxis administration rate. The grey dashed lines represent the 95% (inner) and 99.8% (outer) control limits. Units beyond the outer control limits are deemed outliers. Unit codes are randomized

Figure 2 displays the unadjusted funnel plot for VTE prophylaxis administration rates across the designated BRANZ units. There was considerable variation between units with respect to the proportion of patients receiving prophylaxis, ranging from 48.6% at Unit D to 95.8% at Unit C (Figure S1). The unadjusted funnel plot identified 10 outliers: 6 units as outliers below the mean (Units A, B, D, G, K and L) and 4 units as outliers above the mean (Units C, E, F and H).

**Figure 3.**
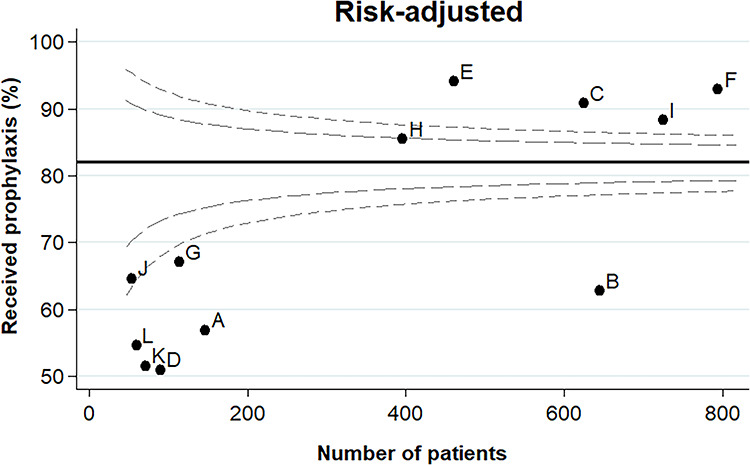
Risk-adjusted funnel plot for venous thromboembolism (VTE) prophylaxis administration rates across Burns Registry of Australia and New Zealand units. Factors included in the risk adjustment were: age; gender; the primary cause of the burn; natural logarithm transformation of the percentage total body surface area burned; whether the patient had a full thickness burn; whether the patient had a documented inhalation injury; whether the patient sustained a burn to their leg and/or foot; whether the patient sustained a burn to their chest and/or trunk; and whether the patient was admitted to the intensive care unit. The black line represents the overall risk-adjusted mean VTE prophylaxis administration rate. The grey dashed lines represent the 95% (inner) and 99.8% (outer) control limits. Units beyond the outer control limits are deemed outliers. Unit codes are randomized

Figure 3 displays the risk-adjusted funnel plot for VTE prophylaxis administration rates across BRANZ units. The risk-adjusted prophylaxis rate ranged from 50.9% at Site D to 94.0% at Site E (Figure S2). The risk-adjusted funnel plot identified 10 outliers: 6 units as outliers below the mean (Units A, B, D, G, K and L) and 4 units as outliers above the mean (Units C, E, F and H). Post-estimation statistics from the mixed effects logistic regression model identified that 32.4% (95% CI, 17.3–52.2) of the variance remained unexplained by the model.

### Association between VTE prophylaxis use and in-hospital outcomes

Table 2 displays the associations between VTE prophylaxis use and the selected in-hospital outcomes. The in-hospital mortality rate was low, with <1% of patients dying. Unit-specific variance in survival was observed (Figure S3). There was no univariate association between receiving VTE prophylaxis and in-hospital mortality. After adjusting for relevant confounding factors, the adjusted odds of in-hospital mortality were 79% lower for patients receiving prophylaxis compared to patients who did not receive prophylaxis (OR = 0.21; 95% CI, 0.07–0.63). The association between VTE prophylaxis and cause of death was not significant; multisystem organ failure was the most common cause of death across both groups (Table S3). The rate of non-fatal VTE events was also low, with 3% of patients experiencing such an event. The frequency of each non-fatal VTE ICD-10-AM code is reported in Table S4. There was a univariate association between receiving VTE prophylaxis and experiencing a VTE; this association did not remain following adjustment for relevant factors. The complete risk-adjusted mixed effects regression model outputs are presented in Tables S5 and S6.

**Table 2:** Association between VTE prophylaxis use and in-hospital outcomes

			**Unadjusted **		**Adjusted **	
			**OR (95% CI) **	***P*-value **	**OR (95% CI) **	***P*-value **
In-hospital Mortality	No	Yes				
No prophylaxis	889 (99.2%)	7 (0.8%)	1.00	0.65	1.00	0.006
Prophylaxis	3771 (99.3%)	26 (0.7%)	0.81 (0.33, 1.99)		0.21 (0.07, 0.63)	
Non-fatal VTE Event*	No	Yes				
No prophylaxis	353 (99.7%)	1 (0.3%)	1.00	0.010	1.00	0.07
Prophylaxis	1984 (96.6 %)	70 (3.4%)	13.75 (1.87, 101.32)		6.73 (0.84, 54.08)	

^*^For patients with International Statistical Classification of Diseases and Related Health Problems, 10^th^ Revision, Australian Modification diagnosis codes accompanying their admissions data

*CI* confidence interval, *OR* odds ratio, *VTE* venous thromboembolism

Units C, E, F, H and I had a risk-adjusted VTE prophylaxis administration rate >80%; units B, J and G had a risk-adjusted VTE prophylaxis administration rate between 60% and 79%; and units A, D, K and L had a risk-adjusted VTE prophylaxis administration rate <60% (Figure 2). There was no association between these three groups and in-hospital mortality or non-fatal VTE events (Table S7).

## Discussion

Burn patients are at a theoretically increased risk of experiencing VTE, yet there are no evidence-based or consensus guidelines for prophylactic management. This inevitably leads to wide variation in VTE treatment practices. Using data from the BRANZ, we demonstrated that although 80% of patients receive some kind of VTE chemoprophylaxis during their admission there is significant variation between Australian and New Zealand burn units with respect to the use of VTE prophylaxis. Approximately a third of this variation could be attributed to site-specific factors. The rate of in-hospital mortality (<1%) and non-fatal VTE events (approximately 3%) were low. The use of VTE prophylaxis was strongly associated with reduced odds of in-hospital mortality.

Our data demonstrates differences in the characteristics of patients who did and did not receive VTE prophylaxis. Specifically, patients who received prophylaxis were older and sustained larger and deeper burns than those who did not receive prophylaxis. Furthermore, a greater proportion of patients who received prophylaxis were admitted to the ICU, sustained an inhalation injury and had a burn injury affecting their leg and/or foot. These differences are consistent with previously identified risk factors for experiencing a VTE event. In their 2011 study using data from the NBR, Pannucci *et al.* identified increasing age, inhalation injury, admission to the ICU and increasing %TBSA burned as risk factors for experiencing a VTE event [7]. The American College of Chest Physicians also list advanced age and extensive or lower extremity burns as potential risk factors for a VTE in burn patients [28, 29]. Therefore, it appears that Australian and New Zealand burn clinicians are prescribing VTE prophylaxis to patients who present with an increased risk of experiencing a VTE event.

Overall, 80% of patients received some kind of VTE prophylaxis during their acute admission. However, the proportion of patients who received prophylaxis at each unit varied substantially, ranging from 49% of patients at one unit to >95% at another unit. This variation in practice remained after adjusting for relevant confounding factors including age, %TBSA burned and burn depth. This finding suggests the absence of a consensus approach to VTE prophylaxis management in Australian and New Zealand burn units. Moreover, these findings are consistent with a published paper in 2011 from Abedi and Papp, who surveyed VTE prophylaxis practices in Canadian burn centres and did not identify a consistent approach or treatment algorithm between burn centres [1]. This variation also suggests that the quality of burn care may vary between contributing burn centres (i.e. VTE prophylaxis administration may act as a surrogate for quality of care). Further investigation of VTE prophylaxis use and hospital-specific protocols on a local scale is required to understand the reasons for this variation (e.g. whether particular units have a larger proportion of patients who have contraindications to chemoprophylaxis, whether there has been a data entry anomaly, etc). Such an initiative may assist in the development of best practice VTE prophylaxis guidelines that can be implemented across Australian and New Zealand burn units to improve patient care. Exploring differences in VTE prophylaxis policy between Australian and New Zealand burn units may also explain the variation between contributing units and assist in the development of best practice VTE prophylaxis guidelines.

The rate of in-hospital death was <1%. This figure is consistent with previous reports involving BRANZ data [20, 21], but lower than reports using NBR data [30, 31]. Contributing units differed with respect to survival rate. Our data provide some (albeit not conclusive) evidence of an association between VTE prophylaxis administration and survival rates at the unit-specific level (i.e. units with lower prophylaxis compliance having higher mortality rates). To the best of our knowledge, our observed finding of decreased mortality following VTE prophylaxis administration in burn patients has not previously been reported. These findings suggest that VTE chemoprophylaxis may reduce the odds of in-hospital mortality following burn injury in adults. However, there was no association between the three administration rate groups and in-hospital mortality. Further information regarding the type, timing and dosage of VTE chemoprophylaxis is required before specific clinical recommendations can be made. Comparisons with other published studies is difficult given the paucity of research undertaken in this area [2, 15, 28, 29]. It is not possible to compare the current study and the NBR study from Pannucci *et al.* as the NBR did not collect data on the use of chemoprophylaxis at that time [7]. Approximately 3% of patients in the current study experienced a non-fatal VTE event. This figure is similar to the 3.2% incidence rate reported by a 2017 single-site Canadian study [15] but is higher than the 0.6% VTE incidence rate reported by Pannucci *et al.* in their analysis of NBR data [7]. This discrepancy may be explained by differences in the sample sizes of the two studies; Pannucci *et al.* included more than 33,000 patients over a 12-year period in their analyses. There was no evidence for an association between VTE prophylaxis use and non-fatal VTE events. This finding is consistent with the aforementioned Canadian study from Sikora and Papp, who concluded that chemoprophylaxis does not prevent VTE in burn patients [15]. However, caution must be noted when making comparisons with the Sikora and Papp study, which was a single-site study involving 26 patients. There was also no association between the three administration rate groups and non-fatal VTE events. These findings suggest that VTE chemoprophylaxis may not prevent non-fatal VTE events occurring in adult burn patients. Again, further research is required to inform any changes to clinical practice.

The limitations of this study must be considered. First, the registry only asks a single question about whether patients received VTE prophylaxis; there are currently no data fields relating to the specific type, dosage or timing of chemoprophylaxis prescribed. Furthermore, the registry currently does not collect information on the use of non-pharmacological thromboprophylaxis measures, such as early mobilization [10] and compression stockings [32]. The absence of additional data relating to pharmacological and non-pharmacological prophylaxis approaches limits our ability to provide additional recommendations for clinical practice. Second, data collectors and clinical coders within different jurisdictions may code similar information differently, meaning that there may be variation within the ICD-10-AM codes entered into the BRANZ. Third, the registry does not collect many of the physiological measurements required to calculate the Acute Physiology and Chronic Health Evaluation score (or other measures of illness severity) and only has ICD-10-AM codes (and therefore CCI weightings) for half of the study population. Consequently, it was not possible to include a measure of underlying illness in the current study. The inclusion of such a measure may have influenced the results, given many underlying illnesses are associated with increased mortality. Fourth, while the registry collects data on cause of death from the medical record it does not have access to autopsy results. A lack of access to comprehensive data on cause of death limits our ability to examine the types of deaths that occur following VTE prophylaxis administration. Fifth, the registry is a deidentified database that does not have linkages with other primary care datasets or outcomes; we were therefore unable to determine how many events or deaths associated with VTE occured in burn patients following discharge from a specialist burn unit. Finally, it is possible for patients to experience a VTE yet remain asymptomatic. It is therefore possible that the true number of patients experiencing a VTE is greater than what is reported here.

## Conclusions

This is the first study to explore variation in the use of VTE prophylaxis in designated Australian and New Zealand burn units and investigate the association between prophylaxis use and in-hospital outcomes. Significant variation in the use of VTE prophylaxis was observed between the units, but prophylaxis use was associated with reduced odds of mortality. These findings provide an opportunity to engage with units to further explore the observed differences in prophylaxis use and develop future best practice VTE prophylaxis guidelines.

## Supplementary Material

VTEProphylaxis_SupplementaryMaterials_V6_B_and_T_revisedV1_tkaa044

## Data Availability

The datasets generated and analysed during the current study are not publicly available, as the authors do not have permission to share these data publicly.
