# Supplementary material for: Venous thromboembolism prophylaxis practice and its association with outcomes in Australia and New Zealand burns patients
Source: Burns Trauma. 2021 Feb 11;9:tkaa044. doi: 10.1093/burnst/tkaa044 (PMC7901708; doi:10.1093/burnst/tkaa044)
Supplement: VTEProphylaxis_SupplementaryMaterials_V6_B_and_T_revisedV1_tkaa044 [file vteprophylaxis_supplementarymaterials_v6_b_and_t_revisedv1_tkaa044.docx]

**SUPPLMENTARY MATERIALS**

| **Supplementary Table 1.** ICD-10-AM codes for non-fatal VTE events | |
| --- | --- |
| *ICD-10-AM Code* | *Diagnosis* |
| I24 | Coronary thrombosis not resulting in myocardial infarction |
| I26 | Pulmonary embolism |
| I63 | Cerebral infarction |
| I74 | Arterial embolism and thrombosis |
| I81 | Portal vein thrombosis |
| I82 | Other venous embolism and thrombosis |
| T81.7 | Vascular complications following a procedure, not elsewhere classified |
| T82.56 | Mechanical complication of vena cava device |
| T82.79 | Infection and inflammatory reaction due to cardiac and vascular devices, implants and grafts, not elsewhere classified |
| T82.82 | Embolism and thrombosis following insertion of cardiac and vascular prosthetic devices, implants, and grafts |
| T82.89 | Other specified complications of cardiac and vascular prosthetic devices, implants, and grafts |
| T82.9 | Unspecified complication of cardiac and vascular prosthetic device, implant, and graft |
| T83.82 | Embolism and thrombosis following insertion of genitourinary prosthetic devices, implants, and grafts |
| T84.82 | Embolism and thrombosis following insertion of internal orthopaedic prosthetic devices, implants, and grafts |
| T85.84 | Embolism and thrombosis following insertion of other prosthetic devices, implants, and grafts |
| Y44.2 | Anticoagulants causing adverse effects in therapeutic use |
| Y71 | Cardiovascular devices associated with unintentional events |
| Y84 | Other medical procedures as the cause of abnormal reaction, or of later complication, without mention of unintentional events at the time of the procedure |
| ICD-10-AM = International Statistical Classification of Diseases and Related Health Problems, 10th Revision, Australian Modification; VTE = venous thromboembolism. | |

| **Supplementary Table 2.** Demographic, event, and injury characteristics | | | |
| --- | --- | --- | --- |
|  | *Invalid response*  *(n = 369)* | *Valid response*  *(n = 4697)* | *p*-value |
| Age, median (IQR) years | 40.0 (26.0-54.0) | 42.0 (28.0-57.0) | 0.051^ |
| Gender |  |  | 0.44 |
| Male | 261 (70.7%) | 3410 (72.6%) |  |
| Female | 108 (29.3%) | 1287 (27.4%) |  |
| Primary cause of burn injury^a^ |  |  | 0.54 |
| Flame | 157 (42.8%) | 2159 (46.2%) |  |
| Scald | 109 (29.7%) | 1263 (27.0%) |  |
| Contact | 49 (13.4%) | 644 (13.8%) |  |
| Other Cause | 52 (14.2%) | 609 (13.0%) |  |
| TBSA, median (IQR)^b^ | 2.0 (1.0-5.0) | 3.5 (1.5-8.0) | <0.001^ |
| FT TBSA, median (IQR)*^c^ | 1.0 (0.5-2.0) | 1.5 (0.8-4.0) | 0.003^ |
| %TBSA Group^b^ |  |  | <0.001 |
| 0-9% | 312 (88.6%) | 3611 (78.4%) |  |
| 10-19% | 27 (7.7%) | 646 (14.0%) |  |
| ≥ 20% | 13 (3.7%) | 350 (7.6%) |  |
| Superficial Burn^d^ |  |  | 0.071 |
| No | 159 (49.4%) | 2304 (54.6%) |  |
| Yes | 163 (50.6%) | 1917 (45.4%) |  |
| Full Thickness Burn^e^ |  |  | <0.001 |
| No | 229 (72.2%) | 2653 (62.8%) |  |
| Yes | 88 (27.8%) | 1571 (37.2%) |  |
| Burn to Chest or Trunk |  |  | 0.010 |
| No | 288 (78.0%) | 3371 (71.8%) |  |
| Yes | 81 (22.0%) | 1326 (28.2%) |  |
| Burn to Legs or Feet |  |  | <0.001 |
| No | 256 (69.4%) | 1892 (40.3%) |  |
| Yes | 113 (30.6%) | 2805 (59.7%) |  |
| Inhalation Injury^f^ |  |  | 0.79 |
| No | 342 (94.5%) | 4398 (94.1%) |  |
| Yes | 20 (5.5%) | 274 (5.9%) |  |
| ICU Admission^g^ |  |  | 0.018 |
| No | 317 (92.2%) | 4126 (87.9%) |  |
| Yes | 27 (7.8%) | 569 (12.1%) |  |
| ICU LOS, median (IQR) hours**^h^ | 57.8 (33.7-114.6) | 67.0 (32.6-253.7) | 0.31^ |
| ICD-10-AM Codes submitted |  |  | <0.001 |
| No | 241 (65.3%) | 2289 (48.7%) |  |
| Yes | 128 (34.7%) | 2408 (51.3%) |  |
| CCI Weight*** |  |  | 0.36 |
| 0 | 108 (84.4%) | 1919 (79.7%) |  |
| 1 | 12 (9.4%) | 329 (13.7%) |  |
| > 1 | 8 (6.3%) | 160 (6.6%) |  |
| Data presented as frequency (percentage) unless otherwise specified. All *p*-values are from a chi-squared test, unless otherwise specified.  CCI = Charlson Comorbidity Index; FT = full thickness; ICD-10-AM = International Statistical Classification of Diseases and Related Health Problems, Tenth Revision, Australian Modification; ICU = intensive care unit; IQR = interquartile range; LOS – length of stay; TBSA = total body surface area.  Data missing for: ^a^24 patients, ^b^107 patients, ^c^160 patients, ^d^523 patients, ^e^525 patients, ^f^32 patients, ^g^27 patients, and ^h^5 patients.  * For patients with a full thickness burn.  ** For patients admitted to the ICU.  *** For patients with ICD-10-AM codes.  ^ *p*-values from Mann-Whitney *U* test. | | | |

| **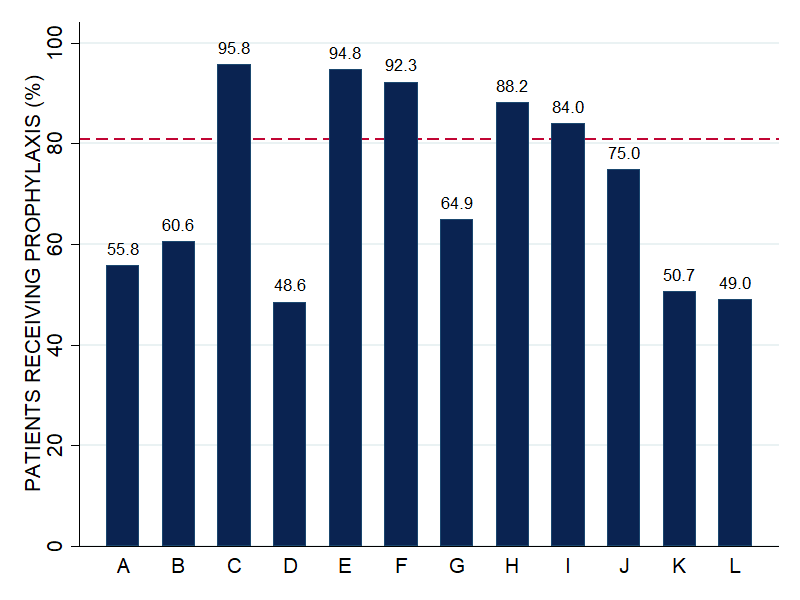** |
| --- |
| **Supplementary Figure 1.** Rate of venous thromboembolism prophylaxis administration by contributing unit. Units were randomly allocated an alphabetical label. The dashed red line represents the mean rate of venous thromboembolism prophylaxis administration across all units. |

| **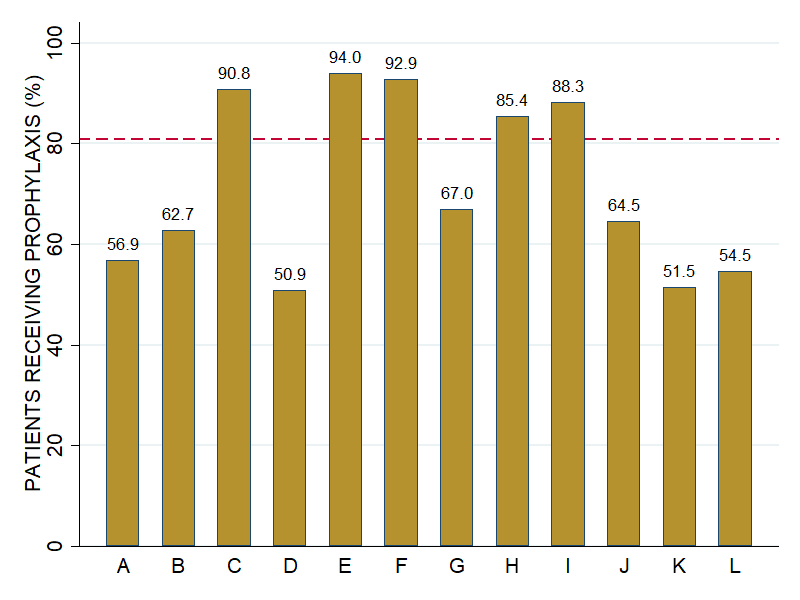** |
| --- |
| **Supplementary Figure 2.** Risk-adjusted rate of venous thromboembolism prophylaxis administration by contributing units. Factors included in the risk-adjustment were: age, gender, the primary cause of the burn, natural logarithm transformation of the %TBSA, whether the patient had a full thickness burn, whether the patient had a documented inhalation injury, whether the patient sustained a burn to their leg and/or foot, whether the patient sustained a burn to their leg and/or foot, whether the patient sustained a burn to their chest and/or trunk, and whether the patient was admitted to the intensive care unit. Units were randomly allocated an alphabetical label. The dashed red line represents the mean rate of venous thromboembolism prophylaxis administration across all units. |

| **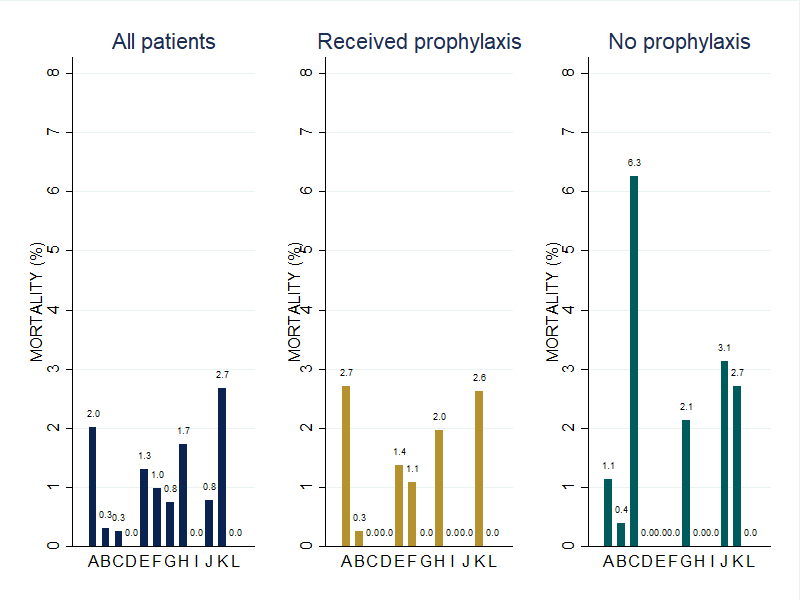** |
| --- |
| **Supplementary Figure 3.** Unit-specific mortality rates. Units were randomly allocated an alphabetical label. Reported percentage is relative to each grouping (i.e., the overall mortality rate at Unit A was 2%, the mortality rate in patients receiving prophylaxis at Unit A was 2.7%, etc.). |

| **Supplementary Table 3.** Cause of death for patients who died in-hospital | | |
| --- | --- | --- |
|  | *No prophylaxis*  *(n = 7)* | *Received prophylaxis*  *(n = 26)* |
| Multisystem organ failure | 5 (83.3%) | 12 (48.0%) |
| Pulmonary (e.g., PE, pneumonia, ARDS) | 0 (0%) | 3 (12.0%) |
| Burns shock | 0 (0%) | 1 (4.0%) |
| Cardiac (e.g., AMI) | 0 (0%) | 1 (4.0%) |
| Renal (e.g., acute renal failure) | 0 (0%) | 1 (4.0%) |
| Sepsis | 0 (0%) | 1 (4.0%) |
| Other cause | 1 (16.7%) | 6 (24.0%) |
| AMI = acute myocardial infarction; ARDS = acute respiratory distress syndrome; PE = pulmonary embolus.  Data presented as frequency (percentage).  Data missing for 2 patients. | | |

| **Supplementary Table 4.** Frequency and percentage of ICD-10-AM codes for non-fatal VTE events | |
| --- | --- |
| *ICD-10-AM Code* | *Number (%)* |
| I24 | 0 (0%) |
| I26 | 6 (0.2%) |
| I63 | 5 (0.2%) |
| I74 | 3 (0.1%) |
| I81 | 1 (< 1%) |
| I82 | 9 (0.4%) |
| T81.7 | 0 (0%) |
| T82.56 | 0 (0%) |
| T82.79 | 1 (< 1%) |
| T82.82 | 1 (< 1%) |
| T82.89 | 0 (0%) |
| T82.9 | 0 (0%) |
| T83.82 | 0 (0%) |
| T84.82 | 0 (0%) |
| T85.84 | 0 (0%) |
| Y44.2 | 4 (0.2%) |
| Y71 | 1 (< 1%) |
| Y84 | 60 (2.5%) |
| * Percentages are relative to the number of patients with ICD-10-AM codes accompanying their admission data.  ICD-10-AM = International Statistical Classification of Diseases and Related Health Problems, 10th Revision, Australian Modification; VTE = venous thromboembolism. | |

| **Supplementary Table 4.** Association between VTE prophylaxis use and in-hospital mortality (multivariable model) | | |
| --- | --- | --- |
|  | Adjusted Odds Ratio (95% CI) | *p*-value |
| VTE Prophylaxis Use |  | 0.006 |
| No (reference) | 1.00 |  |
| Yes | 0.21 (0.07, 0.63) |  |
| Age | 1.08 (1.05, 1.11) | <0.001 |
| TBSA | 1.06 (1.04, 1.08) | <0.001 |
| Was the burn due to a flame? |  | 0.30 |
| No (reference) | 1.00 |  |
| Yes | 1.75 (0.61, 4.99) |  |
| Full thickness burn |  | 0.08 |
| No (reference) | 1.00 |  |
| Yes | 2.34 (0.90, 6.09) |  |
| Did the burn affect the chest or trunk? |  | 0.14 |
| No (reference) | 1.00 |  |
| Yes | 2.08 (0.79, 5.49) |  |
| Inhalation Injury |  | 0.03 |
| No (reference) | 1.00 |  |
| Yes | 3.08 (1.17, 8.54) |  |
| CI = confidence interval; TBSA = total body surface area; VTE = venous thromboembolism. | | |

| **Supplementary Table 5.** Association between VTE prophylaxis use and non-fatal VTE events (multivariable model) | | |
| --- | --- | --- |
|  | Adjusted Odds Ratio (95% CI) | *p*-value |
| VTE Prophylaxis Use |  | 0.07 |
| No (reference) | 1.00 |  |
| Yes | 6.73 (0.84, 54.08) |  |
| Full thickness burn |  | 0.06 |
| No (reference) | 1.00 |  |
| Yes | 1.82 (0.98, 3.39) |  |
| Did the burn affect the chest or trunk? |  | 0.78 |
| No (reference) | 1.00 |  |
| Yes | 1.09 (0.59, 2.02) |  |
| Inhalation Injury |  | 0.72 |
| No (reference) | 1.00 |  |
| Yes | 1.14 (0.56, 2.29) |  |
| ICU admission |  | <0.001 |
| No (reference) | 1.00 |  |
| Yes | 6.37 (3.26, 12.45) |  |
| CI = confidence interval; ICU = intensive care unit; TBSA = total body surface area; VTE = venous thromboembolism. | | |

| **Supplementary Table 6.** Association between VTE prophylaxis administration rate groups and in-hospital outcomes | | | |
| --- | --- | --- | --- |
| *In-hospital Mortality* | No | Yes | *p-value* |
| < 60% administration | 557 (98.9%) | 6 (1.1%) | 0.38 |
| 60-79% administration | 915 (99.6%) | 4 (0.4%) |  |
| ≥ 80% administration | 3188 (99.3%) | 23 (0.7%) |  |
|  |  |  |  |
| *Non-fatal VTE Event** | No | Yes | *p-value* |
| < 60% administration | 151 (96.2%) | 6 (3.8%) | 0.08 |
| 60-79% administration | 420 (98.6%) | 6 (1.6%) |  |
| ≥ 80% administration | 1776 (96.7%) | 59 (3.2%) |  |
| * For patients with International Statistical Classification of Diseases and Related Health Problems, 10^th^ Revision, Australian Modification diagnosis codes accompanying their admissions data.  *p*-values from Fisher’s exact tests. | | | |
